# Gut Microbiota Interaction with the Central Nervous System throughout Life

**DOI:** 10.3390/jcm10061299

**Published:** 2021-03-21

**Authors:** Jorge Ojeda, Ariel Ávila, Pía M. Vidal

**Affiliations:** 1 Neuroimmunology and Regeneration of the Central Nervous System Unit, Biomedical Science Research Laboratory, Basic Sciences Department, Faculty of Medicine, Universidad Católica de la Santísima Concepción, Concepción 4090541, Chile; jojeda@ucsc.cl; 2 Developmental Neurobiology Unit, Biomedical Science Research Laboratory, Basic Sciences Department, Faculty of Medicine, Universidad Católica de la Santísima Concepción, Concepción 4090541, Chile; aavila@ucsc.cl

**Keywords:** gut microbiota, dysbiosis, neurodegeneration, neurodevelopment

## Abstract

During the last years, accumulating evidence has suggested that the gut microbiota plays a key role in the pathogenesis of neurodevelopmental and neurodegenerative diseases via the gut–brain axis. Moreover, current research has helped to elucidate different communication pathways between the gut microbiota and neural tissues (e.g., the vagus nerve, tryptophan production, extrinsic enteric-associated neurons, and short chain fatty acids). On the other hand, altering the composition of gut microbiota promotes a state known as dysbiosis, where the balance between helpful and pathogenic bacteria is disrupted, usually stimulating the last ones. Herein, we summarize selected findings of the recent literature concerning the gut microbiome on the onset and progression of neurodevelopmental and degenerative disorders, and the strategies to modulate its composition in the search for therapeutical approaches, focusing mainly on animal models studies. Readers are advised that this is a young field, based on early studies, that is rapidly growing and being updated as the field advances.

## 1. Introduction

The human body is an ecosystem carrying an incredible diversity of all kinds of microbes that work collectively as a metabolic organ finely tuned and interconnected with host physiology. Microbiota is the ecological community of commensal, symbiotic, and pathogenic microorganisms that literally share our body space [1]. Our gut is one of the most studied organs with abundant microbial activity [2]. In particular, the gut possess approximately 3000 prokaryotic species, majorly composed of Proteobacterias, Firmicutes, Actinobacterias, and Bacteroidetes [3]. Other reasons the gut microbiota have been studied more than microbes in other parts of the body include the fact that stool samples are accepted as a good read-out of the bacteria present in the lining of the gut and are easy to obtain. Gut microbiota perform several functions, such as protection against pathogens, digestion, and production of nutrients, and collaborate in stimulating the immune system [4]. In part, these functions are triggered by metabolites derived from the microbial fermentation of dietary polysaccharides as short-chain fatty acids (SCFAs), of which the majority consist of acetate, propionate, and butyrate [5]. The distinct functions of gut microbiota and its derived metabolites depend of a variety of factors changing in life (e.g., mode of birth delivery, infections, medications, food culture, environmental stressors, life-stage, life-style, and genetic, among others). Changes in the gut microbiota composition alter the brain–gut communication, leading ultimately to the development of different pathologies. In particular, growing evidence supports a role of gut microbiota in the onset of ischemic stroke [6], epilepsy [7], Parkinson’s disease [8], depression, and schizophrenia [9]. The regulation of these pathways and their mechanisms are largely unexplored and the reader should be noted that most of the publications only describe the relationship between gut microbiota dysbiosis and disease, but many open questions remain regarding the causal effect of gut microbiota dysbiosis. Thus, in this review, we summarized selected findings including experiments with human and rodents, whose relevance in vivo for humans might still be a matter of further investigation.

## 2. The Crosstalk between the Gut Microbiota and the Brain

The central nervous system (CNS) consists of the brain and spinal cord, while the peripheral nervous system is formed by the ganglia nerve extended outside CNS and the autonomic nervous system (ANS) that, in turn, comprises sympathetic and parasympathetic branches. The digestive tract has its own nervous system called the enteric nervous system (ENS). In particular, the essential functions of the gastrointestinal tract, such as digestion, absorption, secretion, propulsive movements, and defense, are controlled by neurons from the ENS that connect with the ANS and sensory system (spinal and vagus nerves) and depend on the ability of different cells types associated with the gut wall to integrate distinct cues [10,11]. The CNS, and particularly the brain, displays a bidirectional communication with the gut ecosystem through the physiological control of neurons, hormones, and cytokines [10]. Moreover, the same route provides the CNS with the feedback of gut microbiota adaptations through either metabolites or neurotransmitters production. In parallel, another pathway through which the brain communicates with the gut is the hypothalamic–pituitary–adrenal (HPA) axis [10,12]. This neuroendocrine axis is involved in the emotional influence of the brain on the gut under stress response-associated behavior [13].

In detail, the most direct bidirectional neuronal communication pathway between brain and gut is provided by the cytoplasmic processes in enteroendocrine cells of the small intestine and colon named neuropod cells. These cells elongate in the presence of trophic factors, such as neurotrophins, and are supported by enteric glia to contact a nerve terminal [14]. Additionally, the visceral afferent endings coming from the vagus nerve have chemosensitive receptors that bind hormones and regulatory peptides such as ghrelin, cholecystokinin (CCK), glucagon-like peptide-1 (GLP-1), peptide YY (PYY), and neuropeptide Y (NPY) secreted by enteroendocrine cells (EECs) that influence the regulation of food intake and energy balance [13,15]. On the other hand, intraganglionic laminar vagal afferent endings are positioned in the connective tissue capsule of myenteric plexus ganglia between both muscle layers (longitudinal and circular) to sense and respond to muscle tension produced by both muscle layers [15]. Mice treated with *Campylobacter jejuni* showed that c-Fos protein levels are increased in the viscerosensory (nucleus tractus solitarius) brain region as read-out of its activation and correlate with higher circulating levels of cytokines interleukin (IL)-1, IL-6, and tumor necrosis factor (TNF)-α [16]. Furthermore, the intraduodenal injection of *Lactobacillus johnsonii* reduced renal sympathetic nerves activity and blood pressure and enhanced gastric vagal nerve activity, suggesting that gut metabolites might regulate autonomic neurotransmission via the vagus nerve [17]. In addition, mice supplemented with the probiotic *Bifidobacterium longum* have shown a reduction in anxiety-like behavior associated with chronic colitis. This effect was absent in vagotomized mice [18]. A more recent study demonstrated that fecal microbiota transplantation (FMT) attenuated the effect of chronic mild stress on brain function, evidencing a central link in the brain–gut axis through the vagus nerve [19]. Gut microbiota can modulate gut-extrinsic sympathetic neurons, providing key information about additional circuits of gut-projecting neurons that contribute to better characterization and understanding of how the microbiota–gut–brain axis can be influenced [20]. Of note, we cannot forget that the immune system is an important pathway by which gut–brain–microbiota signaling also occurs [21]. In this regard, the neonatal gut is colonized by maternal vaginal microbiota to stimulate the immune system development [22]. For instance, in germ-free (GF) mice, it was demonstrated that specific species of bacteria from the gut microbiota collaborate in the maturation of innate and adaptive gut immune T cell responses [23,24]. Furthermore, a recent report showed that gut microbiota induces interferon gamma (IFN-γ) secretion by natural killer cells that, in turn, stimulate TRAIL^+^ astrocytes, a specific anti-inflammatory astrocyte subpopulation associated with CNS development [25].

### 2.1. Fundamental Brain–Gut Microbiota Interplay

The bidirectional microbiome–brain communication has multiple effects, some of which orchestrate profound changes in the brain, and is one of the most controversial topics in human health. The microbiota habiting the human body comprises bacteria, yeasts, viruses, parasites, helminths, and protozoa, of which we know the most about the bacterial population within our gastrointestinal tract. Intrinsic factors such as genetic, age, and gender, as well as extrinsic factors such as social situation, exercise levels, antibiotic usage and the consumption of other medications, diet, and pollution, among others, can trigger an imbalance in the interplay between gut microbiota and the brain [26,27,28,29,30,31,32]. In this regard, biopsychosocial disorders and functional gastrointestinal conditions have been correlated with gut dysbiosis [26]. For example, human irritable bowel syndrome (IBS) is frequently related to anxiety, depression, and obsessive-compulsive disorder [33,34,35], as well as schizophrenia and panic disorder [33], whose signs and symptoms include abdominal pain and altered intestinal motility or secretion, all of them controlled by the brain [10,33,36]. Furthermore, clinical effects of gut microbiota linked to brain modulation were observed using oral antibiotics, such as neomycin, paromomycin, and rifaximin, in the treatment of hepatic encephalopathy and other conditions that involve enteric bacteria with the subsequent improvement of neurologic symptoms [37]. The correlation between microbiome–brain communication and behavior has also been observed in experimental animal models. For instance, maternal separation, heat stress, or acoustic stress have all been reported to alter the gut microbiota composition in mice [38]. Moreover, germ-free (GF) mice display an exacerbated hypothalamic–pituitary–adrenal response to stress compared with conventionalized mice. This can either be reversed after monocolonization of *Bifidobacterium infantis* or exacerbated with the enteropathogenic *Escherichia coli* (*E. coli*) at six-weeks postnatal [39]. Additionally, GF mice display a reduction in anxiety-like behavior, with increased motor activity compared with specific pathogen-free (SPF) mice [40,41,42]. These findings correlate with a neurochemical decrease in the expression of N-Methyl-D-aspartic acid (NMDA) receptor subunit NR2B in the amygdala, as well as serotonin receptor 1A in the hippocampus [41,42]. Moreover, GF mice expressed similar levels of brain derived neurotrophic factor (BDNF) to heterozygous BDNF mice [39], and both mice strains have decreased microvilli and widening intercellular space compared with the control group, suggesting similar structural alterations of the intestinal barrier [39,43]. Together, these findings evidence the interplay between gut microbiota and brain manifested through anxiety-related behavior.

### 2.2. Influence of Gut Microbiota on Brain Function

The gut microbiota influences brain neurochemical pathways through different types of cues [5,44]. For example, microbiota has the ability to consume and produce neurotransmitters, such as gamma-aminobutyric acid (GABA), serotonin (5-HT), glutamate, dopamine, and norepinephrine, thus modulating their bioavailability and collaborating with the nervous system in the regulation of distinct functions. In humans, *Bacteroides* ssp, *Parabacteroides*, and *Escherichia* species are GABA producers required for neighboring bacteria, including Pseudomonas, Acinetobacter, and Mycobacterium genera [45]. Furthermore, the relative abundance GABA producer bacteria correlates with traits associated with depression [45]. Axenic mice have reduced levels of GABA in the colon and cardiac plasma, but normal levels in the brain compared with conventional mice, suggesting a strong control of the blood–brain barrier (BBB) to maintain GABA levels [46]. The sensory transducer 5-HT is majorly produced by enterochromaffin cells in closer modulation with gut microbiota by activation of intrinsic and extrinsic primary afferent neurons for peristalsis, secretion, and sensation [47]. Indeed, gut microbiota-derived metabolites increase the peripheral bioavailability of 5-HT by acting on enterochromaffin cells [48]. Moreover, GF mice showed 2.8-fold lower levels of 5-HT in plasma compared with conventional mice [48,49]. One of the potential roles of the serotonergic system is the indirect modulation of lipidic metabolism and intestinal fitness [50]. In this regard, *Turicibacter sanguinis* expresses a sodium symporter-related protein with high homology and identity to the mammalian serotonin transporter (SERT), which participates in the lipid and steroid metabolism by reducing systemic triglyceride levels [50].

Another important neurotransmitter system affected by microbes is glutamate, which is produced by many bacteria including *Corynebacterium glutamicum*, *Brevibacterium lactofermentum*, *Brevibacterium flavum*, and *Lactobacillus plantarum*, among others [51,52]. *E. coli*, *Proteus vulgaris*, and *Bacillus subtilis* produce relatively high levels of dopamine and norepinephrine as a growth factor. However, until now, the systemic pathways that influence this production have been scarcely studied [53].

On the other hand, one of the main gut microbiota chemical signals produced to accomplish their functions are metabolites derived of microbial fermentation of dietary polysaccharides known as SCFAs. SCFAs perform several host functions and their alteration has been related with behavioral disorders, including depression [54], neurodevelopment [55], or anorexia nervosa [56]. SCFAs are absorbed by colonocytes and transported into the portal circulation reaching the body, but mainly in hepatocytes under 100 μM, with the exception of acetate, which is found in the range of 100–200 μM [57,58,59]. It is thought that SCFAs reach the brain because, at least in cell culture, it has been shown that they move across the BBB [60]. Although, another report indicates that only acetate is detected in cerebral spinal fluid (CSF) [61]. Even more, endothelial cells of BBB express SCFAs receptors, such as FFAR3, to protect the BBB by inhibition of pathways related with inflammatory responses through Toll-like receptor 4 (TLR4) and oxidative stimuli [62].

Direct evidence of the effects of SCFAs on the brain is limited. For instance, a small cohort of patients with nerve root block by lumbar radiculopathy showed a negative correlation with the concentration of acetate in CSF compared with the control [61]. Furthermore, mice exposed to a high-fat diet in combination with increased fermentable carbohydrate were treated with radioactive ^11^C-acetate and ^13^C-acetate to evidence that acute acetate administration promotes the expression of regulatory peptides involved in appetite suppression through a reduced catalytic activity of hypothalamic 5’ AMP-activated protein kinase (AMPK) [63]. In the same line, overfed rats exhibited an increased production of acetate, which induced the activation of the parasympathetic nervous system. Moreover, in acetate-infused rats, obesity is abolished after vagotomy or treatment with the parasympathetic blocker atropine [64]. On the other hand, the intraventricular infusion of propionic acid in adult rats induced several alterations in social behavior, sensorimotor ability, and cognition, as well as electrophysiological and biochemical parameters, such as hyperactivity, oxidative stress, and astrogliosis [65,66,67]. The mechanisms by which SCFAs might influence the brain and behavior also involve an inhibitory effect on the histone deacetylase (HDAC) [5]. In this regard, acetylation and deacetylation are the most studied post-translational modification in brain function and disease [68]. Accumulative evidence has shown that increasing histone acetylation after a learning experience promotes memory consolidation. For instance, in rats, the injection of butyrate facilitates associative learning, enhancing long-term potentiation at Schaffer-collateral synapses in the area CA1 of the hippocampus [69]. Following the same line, the daily intraperitoneal and intracerebroventricular injection of butyrate during one month in wild-type mice promoted the inhibition of HDACs, mimicking the effects of environmental enrichment [70]. Additionally, butyrate promotes long-term memory in a contextual fear conditioning and extinction model [69] by neuronal activation of the hippocampal-infralimbic circuit, in which butyrate induces larger and persistent effects on extinction compared with the effects on the formation of initial memory [71]. Moreover, butyrate exerts both neuroprotective and neurogenic effects in cerebral hypoxia-ischemia induced in 7-day-old Wistar rats, by exerting anti-inflammatory effects and positively regulating the expression of trophic factors such as neurotrophins pro-BDNF, BDNF, and nerve growth factor (NGF) [72,73]. In relation with other SCFAs, acetate treatment increases the levels of N-acetylaspartate (NAA) metabolite and adenosine triphosphate (ATP), leading to a significant improvement in motor performance in a traumatic brain injury rat model [74]. This can be partially attributed to the following: (1) inhibition of HDAC activity by acetate, which increases histone acetylation-state [75]; and (2) metabolic response to brain injury that involves acetyl coenzyme A dependent functions such as energy derivation, lipid synthesis, and protein acetylation [76]. The metabolic outcome of acetate is also systemic as a high level of acetate derived from an altered microbiota drives the parasympathetic nervous system activation, promoting β-cell insulin secretion and obesity condition [64]. Furthermore, propionate has shown a metabolic role by stimulating intestinal gluconeogenesis through a gut–brain neural circuit that involves the fatty acid receptor FFAR3 [77]. In turn, mice supplemented for three weeks with a diet enriched in fructo- and galacto-oligosacharides displayed antidepressant and anxiolytic effects, positively influencing stress behavior. Additionally, this prebiotic treatment increased SCFAs in cecal samples, suggesting a link between SCFAs and stress-related behavior [78]. Thus, the known mediators of microbiota–gut–brain communication affected by microbial metabolism can be summarized as SCFAs, neurotransmitters, hormones, and immune system modulators.

### 2.3. The Intestinal Barrier Might Be a Gateway to Drive Neurological Disorders

Overall, an increased intestinal permeability caused by gut microbiota dysbiosis activates an inflammatory response either through the loss of epithelial barrier integrity or the ability of gut microbiota-derived endotoxins (e.g., lipopolysaccharide (LPS)) to cross the epithelial barrier. In rodent models, the intestinal permeability has been observed in stress-related psychiatric disorders [79], with subsequent restoration after probiotic supplementation [80]. Furthermore, a rat model of psychological stress showed that stress-related indicators such as adrenocorticotrophic hormone and norepinephrine were increased in comparison with its control and they are inversely correlated with the intestinal levels of tight junction proteins [81]. Although the studies in humans are limited, they seem to be going in the same direction. For example, increased cortisol levels in acute-stress have been correlated with intestinal permeability in humans [82], suggesting that gut microbiota homeostasis has a protective effect on the integrity of the epithelial cell barrier against pathogens [27,83,84,85]. In particular, SCFAs influence gut health through local effects on intestinal barrier integrity, mucus production, and motility. However, SCFAs’ levels are affected when the number of SCFAs-producing bacteria is decreased, especially in patients with inflammatory bowel disease such as Crohn’s disease, ulcerative colitis, and graft-versus-host disease, among others [86,87,88]. The possible molecular mechanisms of SCFAs action have been addressed in some reports using butyrate treatments and evidencing changes in the expression of genes related to proliferation, differentiation, autophagy, and apoptosis [89,90], as well as genes related to tight-junction [91]. Subsequent to the intestinal barrier integrity, the microbe–host interaction occurs in the mucus along the intestinal epithelium. In this regard, butyrate, as well as acetate and propionate, promotes the expression of genes related to the production of mucin in ileal epithelium of experimental animal models [92,93,94]. In part, this secretory response might be modulated by serotonin via acetate production [95]. Complementarily, it has been reported that mucus is secreted by goblet cells stimulated by *Bifidobacterium dentium*, which secretes metabolites to upregulate the mucin MUC2 and stimulates autophagy-mediated calcium signaling [96].

### 2.4. Gut Microbiota Influences the CNS Though the Modulation of Gut Motility

Similar to the intestinal barrier alterations, the intestinal motility disorders are accompanied by psychosocial manifestations, like stress or depression [97]. In this regard, gut microbiota can directly influence gastro-intestinal motility, affecting the ENS and the smooth muscle control through its derived metabolites. For instance, it has been described that SCFAs in rat distal colon contribute to colonic motility and peristaltic reflex [98,99]. On one side, SCFAs accelerate colonic transit through the vagal control, as demonstrated by pharmacological inhibition with either atropine or vagotomy [98,100]; meanwhile, acetate has been shown to reduce the frequency of spontaneous longitudinal muscle contraction via an inhibitory response by the ENS [99]. However, in both reports, the authors proposed a mechanism that involves nicotinic and 5-HT_3_ receptors, even when they show opposite effects. A possible mechanism of how gut microbiota regulates serotonin production from enterochromaffin cells has recently been reported [101]. In this study, GF mice treated with *Bifidobacterium dentium* showed increased levels of acetate that, in turn, stimulated the serotonergic system in distinct host tissues [101,102]. In an IBS rat model, SCFAs’ supplementation reduced the frequency of colonic activity and increased the contractile amplitude [103]. In humans, the studies are limited by the number of recruited patients and well-control trials. Nevertheless, it has been shown that the intracolonic infusion of undigestible carbohidrates results in the inhibition of gastric tone, an effect that could be mediated by SCFAs [104]. However, patients treated with an intracolonic infusion of SCFAs did not experience changes in intestinal motility [105]. More recent studies showed that delivery of propionate at the colon can induce appetite regulation and metabolic control improvement by stimulating the secretion of PYY and GLP1 [106,107].

## 3. Gut Microbiome and Brain Development

The development of the brain is a long process that begins during the earliest stages of the formation of the embryo and continues through adulthood. In consequence, microbiome dysbiosis, in the mother during intrauterine development or in the offspring after birth, is set to influence the developmental process. In fact, changes in normal gut microbiota have been linked to known actions that affect brain development. That is, depletion of gut microbiota leads to changes in sleep patterns [108] and altered emotional behavior [109]. These effects can subsequently affect the developmental process. However, we begin to understand how gut microbiota can more directly impact brain function. It was thought that bacteria only start colonizing the gut after birth; however, some studies have suggested its presence in the feto-placental unit and amniotic cavity in preterm pre-labor rupture of membranes [110,111,112]. However, prenatal effects are invariably linked to maternal status. Maternal stress and inflammation are one of the most important factors influencing prenatal development [113]. Importantly, it has been shown that this condition can alter the vaginal microbiome in pregnant mice, causing disruption of the developmental process in the offspring [114,115]. More generally, in the absence of environmental challenges, maternal gut microbiome was found to control fetal development, influencing the development of thalamocortical axons, which lead to altered somatosensory behaviors [116]. Remarkably, a later study demonstrates that maternal dysbiosis affects the intrauterine development through altered circulating metabolites, providing unprecedented mechanistic insights into how microbiota can control development [117]. Additionally, in vitro studies have shown that SCFAs, such as propionate and butyrate, can increase neuronal differentiation of neural stem cells [118]. Similarly, adult hippocampal neurogenesis and survival of newly-born neurons are increased in GF mice compared with the control group, suggesting that microbial colonization during early life can affect adult hippocampal neurogenesis and cognitive functions (e.g., spatial learning and memory) [119].

After birth, it is thought that bacteria start colonizing the infant gut and continue to do so up to the point where an adult-like pattern emerges, which is around the third year of life [30,120]. However, a growing body of evidence suggests a slower development of gut microbiota until its mature establishment [121]. Multiple factors influence this process such as maternal race-ethnicity, breastfeeding, mode of delivery, marital status, exposure to environmental tobacco smoke, and indoor pets [122].

Cognitive scores on infants have been associated with the presence of particular bacterial clusters. Moreover, higher diversity at one year of age was associated with lower scores on the early learning test at two years of age [123]. In the long term, the effects of gut microbiome dysbiosis on the development of the brain have been observed by assessing morphological and behavioral outcomes. Specifically, it has been found that commensal bacteria are necessary for normal morphological development and maturation in the gray and white matter [124]. It was also found that myelination was impaired throughout the brain in GF mice. More importantly, GF mice displayed differences in anxiety-related behaviors, in spatial memory, contextual and cued memory, and social novelty, compared with SPF [124]. Increased anxiety was also observed when bacteria were depleted from post-natal day 28 onwards for three weeks, indicating the existence of a developmental window during adolescence conditioning long-term actions of microbiota [125]. These later studies add to a growing body of evidence that describes a GF phenotype where the absence of microbiota has long-term effects on brain function [39,109].

### Neurodevelopmental Disorders Associated with Gut Microbiome Dysbiosis

Gastrointestinal abnormalities have been reported in various neurodevelopmental disorders including cerebral palsy [126], Rett syndrome [127], and autism [128]. Importantly, gastrointestinal abnormalities in autistic patients have been associated with increased intestinal permeability [129] and microbial gut dysfunction [130,131,132]. Beyond that, microbial dysbiosis has been found to vary according to varying severities of autism [133,134]. On the other hand, probiotic treatment of mice with autistic features alters the composition of microbiota and ameliorates autism-specific behavioural abnormalities [130]. More specifically, reconstitution with Bifidobacterium infantis corrected the exaggerated stress response found in GF mice [39]. In a model of maternal inflammation, human commensal *Bacteroides fragilis* corrected gut permeability, changed microbial composition, and improved neurological outcome [130]. Furthermore, based on recent research that has identified maternal metabolites that depend on microbial activity [116], it has been hypothesized that such metabolites could also be part of dietary supplementations aimed to support normal pregnancy [117].

During the first years of an infant’s life, the gut microbiota undergoes different changes (e.g., diversity, functionality, and composition, among others) [135]. This postnatal maturation of the human microbiota is a slow, dynamic, and long process that occurs, at least, within the first decade of life [121]. It represents a crucial stage for the stimulation of the immune system and how it is prepared to protect our body. It has been suggested that, in humans, gut dysbiosis may contribute to deficits in growth and development [136]. The evidence demonstrates that a good way to establish a mature microbiota plays an important role in allergic diseases such as food allergy [137] or asthma [138,139], even though it could be irrelevant in the cognitive development of children [140]. In this regard, one study showed that three-year-old children with established allergic diseases have a reduction of butyrate producing bacteria and an altered IgE-mediated response [141]. Moreover, infant exposure to different external agents, such as antibiotic treatment, pets, siblings, and solid food, has been shown to alter microbiota composition, and has been related to the development of schizophrenia [113]. In this regard, the preliminary results from a study in autistic children treated with the antibiotic vancomycin showed that eight out of ten children had a short-term improvement in autistic symptoms [142]. Although the improvements were not long-lasting, and the authors of the study do not suggest this protocol as a therapy; these results suggest that postnatal changes in gut microbiota might influence disease progression.

## 4. Gut Microbiota Adaptations during Aging

Primary aging is an inevitable process that involves adaptive changes of all body systems to sustain physiological functions compatible with a healthy well-being and quality of life [143,144]. Along life, an accumulative multiplicity of factors at molecular, cellular, and systemic levels determine the performance of the elderly population. Underlying these factors, the gastrointestinal environment of gut microbiota experiences physical changes to promote functional modifications such as the loss of intestinal absorption surface, mucosal immunosenescence, and disturbed motility [145,146,147]. Direct consequences of these alterations in elderly patients are a small bowel bacterial overgrowth [148,149,150,151,152], as well as a lower total bacteria diversity and abundance, which is consistent with decreased gut microbiome intrapersonal variation [30,153,154,155]. In aging, the intestinal microbiota loses the stability reached during adulthood to give way for an imbalance that exacerbates age-related disease [9,156,157]. The onset of aging-related gut microbiota changes and its core composition, either intra or interindividual, are specific; complex; and dependent on each subject, socio-economic status, habits, culture, and geographic location, among other factors [30,157,158] (Table 1). Thus, elderly patients evidenced greater interindividual variability of both Bacteroidetes and Firmicutes in a wide range of their proportions [156]. Studies of bacterial taxa in a small cohort of patients by viable cell count and 16S rRNA sequencing from fecal samples indicated a high number of enterobacteria in adults compared with children [159]. This finding is confirmed in subsequent reports [153,160]. In contrast, it has been described that Bacteroides-Prevotella is reduced [153,160], even though the phylum bacteroidetes and relatives are dominant in approximately 57% of the elderly population [156,161,162]. Moreover, the phylum *Firmicutes* accounts for the 40% of gut microbiota with a distinct pattern of *Clostridium* groups compared with younger adults [156,161]. In detail, it has been described that more than 50% of gut microbiota in the elderly population is represented by the coabundance of *Bacteroides*, *Alistipes*, and *Parabacteroides*, while these genera appear in only 27% of younger adults. Moreover, studies of co-occurrence describe that Bacteroides, Lachnospira, Roseburia, and Dialister are the most prevalent genera among all age groups [162]. During late adulthood, the major gut microbiota changes in centenarians are a decrease in *Faecalibacterium prauznitzii* and the increase in *Eubacterium limosum* [163], while, in semi-supercentenarians (people over 105 years old), the microbial ecosystem is enriched in Akkermansia, Bifidobacterium, and Christensenellaceae [162]. However, centenarians and semi-supercentenarians shared similar relative abundance of *Bacteroidaceae*, *Lachnospiraceae*, and *Ruminococcaceae*, indicating that gut microbiota tends to remain stable between both age groups [162]. Another trend observed with aging is an increased contribution of subdominant families [162]. For instance, in semi-supercentenarians, the *Osillospira*, *Odoribacter*, and *Butyricimonas* showed a positive correlation with age [162]. On the other hand, the genus Akkermansia has been linked with healthy microbiomes and intestinal integrity [164,165,166]; however, in elderly mice (mean of 28 months old), it was almost absent, while more *Rickenellaceae*, *Lachnospiraceae*, *Ruminococcaceae*, and *Clostridiaceae* were detected in comparison with younger or adult mice [164]. In particular, the most consistent overrepresented genus between elderly humans and mice is the genus *Alistipes* [164].

### 4.1. Gut Microbiota in Frailty Syndrome

During the temporal progression of aging, older adults experience a decline in the state of functioning with the subsequent apparition of distinct pathologies, among which the most common is the frailty syndrome. This geriatric condition carries an increased vulnerability of health, disability, hospitalization, and death [167]. Frailty condition correlates with the loss of gut microbiota diversity [164,168]. Analysis of co-abundance groups of bacteria reveals that the transition from healthy elderly to frailty subjects involves an increase of *Prevotella* and *Ruminococcus* as well as *Alistipes* and *Oscillibacter* [169]. Subsequently, elderly patients with severe frailty syndrome have shown a reduced number of *Bacteroides/Prevotella*, *Eubacterium rectale/Clostridium coccoides*, and *Faecalibacterium prausnitzii* [160,168], while *Ruminococcus* and *Atopobium* were increased [160]. A recent report in a cohort of 728 female twins indicates that among the species more abundant in patients with frailty syndrome are *Eubacterium dolichum* and *Eggerthella lenta* [168]. Thus, the characterization of gut microbiome and its association with frailty syndrome could represent an important way for a possible therapeutic intervention to relieve the effects of this condition (Table 2).

### 4.2. Gut Microbiota on Immunosenescence

The gut microbiome experiences changes associated with the alteration of several functions, such as protection against pathogens, digestion, and production of nutrients, among others [4]. Another profound alteration linked to the gut microbiota during aging is the immunosenescence (Table 2). The cellular and serological responses from the immune system decline with age, and this is evident in the increased prevalence of infectious diseases, autoimmune disorders, and gut microbiota alterations, among others [170]. During aging, especially in the frailty syndrome, the subjects reach a systemic inflammatory status that causes an altered immunological response of B and T cells. This results in an increased sensitivity to infections [171] when the microbiota is disturbed [172,173]. Furthermore, antibodies against commensal gut microbiota are also increased in elderly people [174]. In particular, elderly subjects have shown higher levels of TNF-α, IL-12, IL-6, and IL-8 [175,176,177,178]. In this regard, TNF disruption in aged mice, either by genetic depletion or pharmacological blockage, increases survival and attenuates inflammation [176]. Furthermore, TNF KO mice treated with Lipopolysaccharides (LPS) have normal IL-6 levels and its inflammatory response is similar to young mice [177]. Interestingly, seniors and centenarians manifest an inflammatory status evidenced by the correlation of the circulating cytokines IL-6 and IL-8 with a reduction in the population of some butyrate producing bacteria, such as *E. rectale*, *E. hallii*, and *E. ventriosum* [163,169]. This could be partially explained by the presence of the bacterial cell wall component muramyl dipeptide in the circulatory system, thus providing a piece of evidence pointing to increased intestinal permeability [177,179]. Along this line, axenic mice have shown less paracellular intestinal permeability compared with old wild type mice [177]. However, colonized GF mice with faecal microbiota derived from old mice evidenced a high level of intestinal permeability in comparison with GF mice colonized with microbiota derived from young mice, suggesting that dysbiosis of old mice causes an increased paracellular permeability and inflammatory systemic status. Supporting this idea, old GF mice are protected from age-associated inflammation and elevated levels of IL-6 even under LPS treatment [177]. Thus, the functional crosstalk of the immune system and gut microbiota is essential to avoid profound negative effects on human aging.

**Table 1:** Phylum changes during aging.

	Increase	Decrease	Unaltered
**Reported gut microbiota changes during aging**	**Phylum Firmicutes:**	**Phylum Firmicutes:**	**Phylum Firmicutes:**
	*Clostridium difficile* *Clostridium cluster IV* [161,169] *Clostridium cluster IX* *Faecalibacterium spp* [169] *Enterococcus* gender *Ruminococcus gender* *Eubacterium limosum* *Oscillibacter* gender *Lactobacillus* [175] *Lachnospiraceae*	*Clostridium cluster IV* [155] *Faecalibacterium spp* [175] *Lactobacillus*] [160] *Faecalibacterium prausnitzii* *Eubacterium rectale* *Eubacterium hallii* *Eubacterium ventriosum* *Blautia coccoides*	*Clostridium clostridiforme*
	**Phylum actinobacteria:**	**Phylum actinobacteria:**	
	*Atopobium* gender	*Bifidobacteria* gender	
	**Phylum Bacteroidetes:**	**Phylum Bacteroidetes:**	
	*Bacteroides* gender *Alistipes* gender *Parabacteroides* gender	*Prevotella*	
	**Phylum Proteobacteria:**		**Phylum Proteobacteria:**
	*Enterobacteriaceae*		*Desulfovibrio spp*

**Table 2:** Microbiota alterations during age-associated conditions.

	Increase	Decrease
**Frailty Syndrome**	*Prevotella* *Ruminococcus* *Alistipes* *Oscillibacter* *Eubacterium dolichum* *Eggerthella lenta*	*Prevotella* *Clostridium coccoides* *Faecalibacterium prausnitzii*
**Immunosenescence**		*Eubacterium rectale* *Eubacterium hallii* *Eubacterium ventriosum*
**Behavioral alterations**	*Porphyromonadacea*	*Bifidobacterium breve* A1 *Lactobacillus* *Bifidobacterium longum*
**Locomotor decline**		*Prevotella* *Barnesiella*
**Nutritional status**		*Ruminococcus* *Lactobacilli* *Bifidobacteria*
**Geriatric** **infections**	*Clostridium difficile*	*Bifidobacteria* *Bacteroides* *Clostridium leptum* *Blautia coccoides*

Note. Depending of the literature, the same genus bacteria can be reported as increased or decreased.

### 4.3. Influence of Gut Microbiota on Aging-Associated Pathologies

The frailty syndrome, diet unbalance, and inflammation are influenced by gut microbiota and, in turn, they have been linked with an impaired cognitive function in the elderly population [10]. More specifically, it has been observed that aged mice have shown anxiety-like behaviors that correlate with increased gut permeability and abundance of the Porphyromonadacea bacterial family [178]. Interestingly, Porphyromonadacea has been previously related to aging-associated diseases, such as depression [180], inflammation [181], and cognition [181]. The potential effect of gut microbiota on the maintenance of cognitive functions in elderly subjects has been assayed through the probiotic supplementation of *Bifidobacterium breve* A1 [182], which has been shown to prevent cognitive impairment in a mouse model of Alzheimer disease [183]. Moreover, in a cohort of 117 subjects, it was observed that supplementation with *B. breve* 1A improves the score of immediate memory tested in neuropsychological trials [184]. In mice, probiotic supplementation with *Lactobacillus casei* LC122 or *Bifidobacterium longum* BL986 promoted both muscle strength and function, as well as improvement of the integrity and function of the gut barrier [185]. Surprisingly, probiotic supplementation also improved learning and memory, apparently through an increased expression of neurotrophic factors in the hippocampus [185]. Of note, the analysis of cognition, memory, anxiety, or any other behavioral parameter is dependent on the locomotor activity, which is reduced during aging in mice [178,185].

### 4.4. Gut Microbiota in Locomotor Decline during Aging

In aging, movement and strength evidence a clear decline, caused, in part, by alterations in the balance of energy metabolism, nutrition, hormonal influence, inflammation, sedentary, loss of physical activity, repetitive movements, neuromuscular conditions, muscle weakness, and sarcopenia, which increase the risk of developing age-associated locomotor diseases. Recent evidence has shown that preservation of gut microbiota can influence the neuromotor axis [186,187]. For instance, FMT from older humans classified as high-physical-functioning in adult GF mice improved muscle strength in the colonized mice compared with the fecal transfer from low-functioning older adults [188]. In this regard, *Prevotellaceae*, genus-level *Prevotella* and *Barnesiella*, might influence the maintenance of muscle strength [188] and prevent either physiological decline of musculoskeletal function [189] or the progression of frailty syndrome [160,169,190]. In addition, butyrate treatment has been shown to increase muscle fiber cross-sectional area (CSA) as well as to prevent the intramuscular accumulation of fat in 26-month-old female mice [191,192]. Moreover, butyrate can promote the expression of muscle markers in response to physiological loss of muscle innervation [191]. In this regard, recent novel insights have shown that GF mice display reduced locomotor and muscle strength compared with the control group [193]. Interestingly, GF mice presented low quantities of the substrate for acetylcholine neurotransmitter, choline, in the serum [193], which is involved in locomotor activity. Furthermore, tibialis anterior muscles of GF mice have shown a reduced expression of the AChR subunits and genes that codified for AChR-scaffolding proteins [193], suggesting a role for gut microbiota-derived metabolites in the peripheral nervous system [194]. Thus, consumption of probiotics and prebiotics to modulate gut microbiota composition might be used as a potential treatment approach in age-related disease and sarcopenia [166,191,195,196].

### 4.5. Gut Microbiota in Nutritional Status in Aging

Nowadays, the diet of the elderly population has gained more attention because this is the main controllable aspect of the gut microbiota environment that can exert a positive impact supporting healthier aging [169]. Moreover, pro and prebiotics have emerged as a prominent alternative for the dietary industry to relieve aging-associated diseases [197,198]. Diet has been proposed as a modulator of gut microbiota in different conditions, such as rheumatoid arthritis, osteoarthritis, epilepsy, Parkinson’s disease (PD), and Alzheimer’s diasease (AD), among others [199,200,201,202,203]. Interestingly, in elderly subjects defined as community dwellers, a direct correlation was found between gut microbiota diversity and a diet rich in fibre and low in fat compared with elderly subjects that live long-term in residential care [169]. In contrast, gut microbiota diversity was reduced when an elderly individual ingested a moderate or high-fat diet with a low proportion of dietetic fibre [169]. These results suggest that a balanced diet, accompanied by daily physical activity, can support a healthier gut microbiota. In fact, high levels of butyrate have been shown to correlate with fit subjects [204]. Furthermore, levels of butyrate, as well as acetate, propionate, and valerate, were higher in elderly subjects classified as community dwellers in comparison with elderly subjects that live in long-term residential care [169]. These results were also supported by a metagenome analysis where the levels of enzymes that participate in the synthesis of these metabolites were increased [169], even though other studies reported a shift reduction of SCFAs [175] and its producers [162]. Regarding a high production of SCFAs during aging, Claesson and co-workers have suggested *Ruminococcus* [169], whose expression was elevated [160], as a potential candidate.

A recent study described the association between gut microbiota and vitamin D [205]. In particular, approximately 600 older men showed a correlation of high levels of 1,25(OH)2D, the active form of vitamin D, with greater α-diversity of microbes and butyrate-producing bacteria, which is linked with good gut microbial health [205]. In aging mice, metagenomic studies of gut microbiota have revealed less biosynthesis of vitamin B12 and B7, as well as an overabundance of genes to process monosaccharides [164]. In contrast, the carbohydrates fructooligosaccharides and raffinose, which promote the growth of *Lactobacilli* and *Bifidobacteria*, were decreased [164]. It is worth noting that both genera are the most widely used in probiotics [206,207,208].

### 4.6. Gut Microbiota in Prevalent Geriatric Infection

In aging, gut microbiota alteration commonly goes along with an increased prevalence of gastrointestinal infections [159]. It has been described that infection by *Clostridium difficile*, a common healthcare-associated infection that causes intestinal inflammation and diarrhea, showed high prevalence in long-time elderly hospitalized patients under antibiotic treatment [209]. In particular, a comparative microbiological study showed that 30% of elderly people (over age of 60 years old) are affected by *Clostridium difficile* [209] and, moreover, this infection presents a high frequency in non-hospitalized elderly patients compared with young patients [209,210]. Consistent with the gut microbiota imbalance, elderly patients that present *C. difficile* associated-diarrhea showed a reduced diversity of species, in particular of bifidobacteria [155,159] and bacteroides [159]. Furthermore, reduced levels of Clostridium leptum and Blautia coccoides correlated with the incidence of *C. difficile* [175]. Another difficulty of *C. difficile* infections is the reinfection. In this regard, the development of novel antibiotics such as fidaxomicyn or rifaxamin goes in line with potentiating the antimicrobial effect and clinical response against *C. difficile* [211,212]. A more successful approach to cure *C. difficile* infection is suggested by the results of FMT [213,214]. FMT has been recommended with more than one recurrent severe episode of *C. difficile* infection [215] and it has been demonstrated that it cures nearly 100% of the patients [213,215,216,217].

In summary, strong evidence demonstrates the gut microbiota plasticity during aging and its impact on the health of elderly people (Table 2). Of note, the available literature is descriptive in part, because the integration of cutting-edge approaches (e.g., proteomic, metagenomic, and metabolomic) is still at an initial point. We expect that in a short amount of time they will reveal novel insights into the specific mechanisms by which gut microbiota and its metabolites influence the aging processes.

## 5. Gut Microbiota on the Progression of Degenerative Pathologies

A summary of the current literature about the role of the microbiome on selected neurodegenerative pathologies (PD, AD, multiple sclerosis (MS), amyotrophic lateral sclerosis (ALS), and spinal cord injury) is presented hereunder.

### 5.1. Gut Microbiome on Neurodegenerative Amyloid Disorders

PD is a neurodegenerative disorder characterized by motor symptoms linked to loss of dopaminergic neurons in the substantia nigra. Fecal gut microbiota differs between patients with PD and healthy controls, with reduced concentrations of fecal SCFAs [218,219]. Similar findings have been reported using animal models of the disease [220], where some specific bacterial strains have also been suggested as responsible for either driving [221] or ameliorating PD-like pathology [222]. In PD patients, microbiome analysis has shown that abundance of Enterobacteriaceae and *Helicobacter pylori* is positively associated with the severity of certain PD symptoms [223]. The exact mechanism of gut dysfunction in PD patients is not fully understood. However, the first association between the gut and PD is known as the Braak’s hypothesis [224], which suggests that PD initiates in the gut, before affecting the brain and the symptoms are noticeable. Of note, one study suggested that individuals who have undergone vagotomy present a lower risk to develop PD as they age [225]. Recently, it has been shown that inoculation of the duodenal intestinal wall with αSyn preformed fibrils disrupts the enteric nervous system (ENS) physiology, triggers the formation of αSyn aggregates in the brainstem, and leads to the development of sensorimotor deficits in aged mice, supporting the hypothesis of gut-to-brain progression of αSyn aggregation via the ENS [226].

However, the current evidence in humans remains inconsistent, with reports of both replication and contradictory findings across studies, mainly attributed to methodological differences and patients’ heterogeneity (discussed in more detail [227]). For example, some studies have shown changes in alpha diversity (e.g., either increased or decreased), whereas others do not find differences in this parameter [227,228]. Similarly, the effects of some antiparkinsonian drugs on gut microbiota have also been questioned [228,229,230], thus puzzling the role of gut dysbiosis in human PD pathology and making difficult a straight forward translation from animal model results to the clinic.

The use of experimental animal models has shown that impairment of the bidirectional gut–brain interaction might partially contribute to the early gut disturbances observed in PD patients [231]. Nigrostriatal dopaminergic lesion induced by injection of 6-OHDA in the nigra has led to decreased acetylcholine, total choline levels, and dopamine D1 and D2 receptors, while dopamine levels were increased in the colon. Gastrointestinal inflammation led to reduced dopamine and tyrosine hydroxylase expression in the nigra substance. However, the authors did not assess whether gastrointestinal inflammation per se may trigger PD-like symptoms in this animal model [231]. A recent study in PTEN-induced kinase-1 (Pink1)-deficient mice, a model of PD based on deficiency of Pink-1, a mutated protein associated with autosomal recessive parkinson, has shown that PD-like symptoms can be developed after gastrointestinal infections in mice [232].

In a PD mouse model, where human αSyn is overexpressed, depletion of gut microbiota reduced some motor deficits, SCFA production, and the presence of αSyn aggregates in the brain. The authors suggested that gut microbiota may either promote αSyn aggregation and/or prevent the clearance of insoluble protein aggregates in this mouse model. This hypothesis has been further supported by the discovery of a pathogenic strain of *Escherichia coli*, which is capable of producing an amyloid protein that is able to promote αSyn aggregation [233] in the gut and the brain of experimental animal models [233], as well as enhance PD-like motor deficits in a mouse model of αSyn overexpression [234]. Of note, non-genetically predisposed mice to αSyn overexpression do not develop PD-like motor symptoms, which suggests that the presence of this bacterial strain is not sufficient to promote the disease [234]. Furthermore, mice receiving microbiota derived from either PD patients or PD mice displayed increased motor deficits compared with mice receiving healthy-derived microbiota transplants [8,235]. Additionally, a PD mouse model receiving FMT from healthy mice showed improved motor function, increased striatal neurotransmitters, and reduced neuroinflammation, suggesting that gut microbiota contribute to behaviour and PD pathology in mice potentially via the TLR4-TBK1-NF-κb-TNF-α signaling pathway [235].

Another line of research points towards the role of the gut microbiota in the production of neurotransmitters, such as catecholamines, whose levels are altered in PD, AD, and other major depressive disorders [236]. Moreover, it has been reported that the gut microbiota may interfere with the effectiveness or availability of pharmacological treatment of these two neurodegenerative conditions. For example, levodopa, the primary treatment of PD, can be metabolized to dopamine by gut microbiota in rats [237], mice, and humans [230]. This conversion to dopamine is mediated by *Enterococcus faecalis* and *Eggerthella lenta* [230], reducing the levels of levodopa in the circulation and reducing the therapeutic efficacy of pharmacological PD treatment.

Similarly, gut dysbiosis has also been reported in both humans [219,238,239] and mouse models of AD [240]. For example, in mild AD patients, a decreased bacterial diversity has been reported (e.g., Firmicutes and *Bifidobacterium*) [239]. A recent study using mendelian randomization has shown a potential protective association between increased abundance of *Blautia* and the risk of developing AD. Specifically, the authors suggested that gut metabolite GABA was associated with a lower risk of AD [241].

In mice receiving a broad-spectrum antibiotic treatment, FMT from senescence-accelerated mouse resistant 1 (SAMR1) improved cognitive function and α- and β-diversities of bacterial species. However, FMT from senescence-accelerated mouse prone 8 (SAMP8), commonly used as a mouse model of AD, did not affect either cognitive function or gut microbiota diversity in broad-spectrum antibiotic-treated mice, suggesting that gut microbiota composition is involved in cognitive dysfunction in AD [242]. In another AD mouse model, it has been proposed that gut microbiota abundance triggers peripheral immune cell infiltration to the brain. Specifically, changes in abundance correlated with the alterations in the frequency of Th2 and CD45^low^CD11b^+^CX3CR1^+^Siglec-H^+^CD206^+^ microglial cells at the early stages, whereas alterations of Th1 and CD45^low^CD11b^+^CX3CR1^+^Siglec-H^+^CD86^+^ microglial cells at late stages [240]. Depletion of gut microbiota blocked Th1 cell infiltration and CD45^low^CD11b^+^CX3CR1^+^Siglec-H^+^CD86^+^ microglial cell activation in AD mice [240], thus, attenuating neuroinflammation, Aβ plaque deposition, and progression of AD.

### 5.2. Gut Microbiome on Spinal Cord Disorders

Multiple sclerosis (MS) is an autoimmune disease characterized by demyelination and axonal damage in the brain and spinal cord. One piece of evidence supporting the role of gut microbiota in MS development came from a study in monozygoctic human twin pairs discordant for MS. FMT from MS-affected twins to GF mice led to a higher incidence of experimental autoimmune encepahlomyelitis (EAE) compared with the GF mice colonized with healthy twins derived microbiota [243]. Additionally, changes in bacterial composition and abundance between MS and healthy controls have been reported (e.g., *Psuedomonas*, *Mycoplana*, *Haemophilus*, *Blautia*, *Dorea*, *Methanobrevibacter*, *Akkermansia*, and *Butyricimonas*) [244,245]. These changes in microbiota are dynamic, influenced by the status of immunomodulatory therapies in treated versus untreated MS patients [245], as well as increased Th17 cell frequency in the small intestine [246]. Additionally, a recent study presented gut microbiota-specific IgA^+^ cells as a mediator of MS [247]. These IgA^+^ cells are able to access the CNS during EAE in mice, and can attenuate disease in an IL-10-dependent mechanism by reducing neuroinflammation [248], suggesting a potential mechanism by which gut microbiota can modulate host immunity. Remarkably, it has been suggested that gut microbiota dysbiosis in MS patients is associated with alterations of the ratio SCFA/medium-chain fatty acids [249]. This alteration has been associated with and increased Th1 and Th17 and suppression of regulatory T cells (Tregs) [250].

EAE is the animal model that mimics aspects of MS in humans. GF mice do not develop spontaneous EAE, suggesting that gut microbiota is required for EAE induction [251]. A similar result has been observed following oral antibiotic administration in EAE mice, where either disease severity has been reduced [252] or the onset of mean clinical score has been delayed [243]. This is mediated by the so-called gut–immune axis, through decreasing levels of IFN-γ, IL-17A [251,252,253], IL-10, and TNF-α [252,253], and augmenting the number of Tregs [252]. Dietary interventions, which shape the gut microbiota, have shown promising results in ameliorating EAE in mice. In this case, increased Treg numbers and reduced Th1 and Th17 cells were associated with phenotype [254]. Similarly, IL-17A/F deficient mice have a reduced susceptibility to EAE. Reintroduction of IL-17A into the gut of IL-17 deficient mice re-established their susceptibility to EAE [255].

Amyotrophic lateral sclerosis (ALS) is another spinal cord disorder, characterized by the premature death of motor neurons. The gut microbiota has been included in the list of disease-modifying factors that contribute to the pathogenesis of ALS, with an upregulation of *Bacteroidetes* and down-regulation of *Firmicutes* in ALS patients compared with healthy controls [219,256,257]. Additionally, the levels of butyrate-producing bacteria are lower in ALS patients compared with the control group [258]. However, in another study, patients with ALS did not show changes in the microbiota, compared with the control group [259]. Partially, this discrepancy could be attributed to methodological variations, differences in clinical phenotype, and heterogeneity of the patients. In a mouse model of ALS, gut microbiota dysbiosis contributed to exacerbating disease progression (e.g., *Akkermansia muciniphila*, *Ruminococcus torques*, and *Parabacteroides distasonis*) by favoring the degeneration of motor neurons [260]. Butyrate treatment improved gut integrity and dysbiosis, delaying the onset of ALS in mice [261].

### 5.3. Gut–Brain Axis on Traumatic Spinal Cord Injury

Traumatic spinal cord injury (SCI) is characterized by loss of motor and sensory function due to the spinal cord insult. In a mouse model of traumatic SCI, gut dysbiosis exacerbated lesion size and neuroinflammation within the spinal cord, impairing neurological function. This situation was partially reverted after feeding SCI mice with probiotics enriched with lactic-producing bacteria for 5 weeks following injury [262]. In this study, major changes were reported in Bacteroidales and Clostridiales, whereas minor changes occurred in Anaeroplasmatales, Turicibacterales, and Lactobacillales [262] bacterial taxes. Treatment with probiotics enriched with lactic-producing bacteria increased the number of regulatory T cells, a subpopulation of T cells that play a key role in maintaining immune homeostasis and remodelling processes [263], in the mesenteric lymph nodes, leading to a reduction in inflammation within and outside the CNS [262]. A second study, using a rat model of traumatic SCI, also found alterations in *Lactobacillus intestinalis*, *Clostrodium disporicum*, and *Bifidobacterium choerinum* [264]. Unlike Kigerl et al. [262], microbiota changes in SCI rats were analyzed at 8 weeks following injury, suggesting that gut dysbiosis is not a transient phenomenon.

Gut dysbiosis is not only developed in animal models of traumatic SCI, but also in SCI patients [265,266]. In a small cohort of SCI patients, 16rRNA sequencing from fecal samples showed a reduction in butyrate-producing bacteria compared with healthy controls [265]. It has been suggested that gut dysbiosis following traumatic SCI develops as a secondary consequence to the loss of autonomic control over the gastrointestinal tract [267]. Additionally, a recent study has shown that the spinal level at which the injury occurs (i.e., cervical versus thoracic) will induce different changes in gut microbiota composition [266].

## 6. Therapeutic Implications

**Figure 1:**
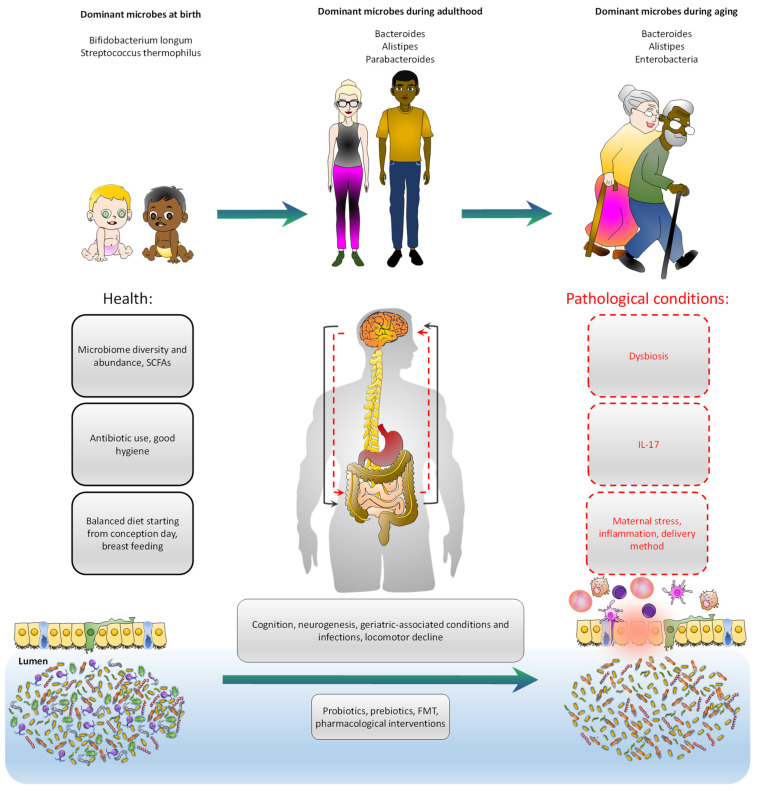
**Gut microbiota in health and disease**. The gut–brain communication contributes to maintaining a healthy status, which is mainly promoted by some of the factors mentioned on the left side (microbiome diversity and abundance, production of SCFAs, among others). The gut microbiota composition is affected during pathological conditions by some of events mentioned on the right side in red color (dysbiosis and gut produced cytokines, such as interleukin (IL)-17, among others). The relationship between the gut and the brain affects different functions in our body throughout life, such as cognition, neurogenesis, locomotor decline, and so on. The integrity of the gut is disrupted during pathological conditions, increasing intestinal permeability and recruitment of immune cells (right side). In the same line, the composition of dominant microbes during life varies. The use of probiotics, prebiotics, and FMT, among others, has been shown to modulate brain-gut communication, with an optimal time window during postnatal development. SCFAs: short-chain fatty acids, FMT: fecal microbiota transplantation.

Over the last decade, the role of gut microbiota in human physiology has become more and more evident (Figure 1), not only affecting disease progression, but also as a modulator of drug availability following pharmacological treatment [268]. In this regard, substances that can positively affect the microbiota–gut–brain axis have emerged as potential therapeutic targets. Among them, we can find probiotics, prebiotics, synbiotics, postbiotics, diet, FMT, and pharmacological approaches. All of them provide promising evidence to suggest that they positively impact gut-related comorbidities [269] and cognition [270].

In terms of probiotics, it is important to mention that their benefits are both strain- and time-period dependent, and not all probiotics will have a beneficial effect [270,271,272,273]. In a randomized controlled trial in AD patients, a multistrain probiotic in a milk drink resulted in improvements in cognition [270], whereas using other strain compositions did not affect cognition [273]. Moreover, in MS patients, oral administration of a probiotic containing *Lactobacillus*, *Bifidobacterium*, and *Streptococcus* was associated with either an enrichment of genera depleted in MS or a decrease of genera associated with dysbiosis in MS [274]. Potential probiotic candidates, which might have proven benefits in preclinical animal models, do not have a guaranteed successful translation into human studies [271]. The limitations of probiotics include their daily consumption to maintain positive effects (e.g., the bacterial strains normally do not become residents in the gut), ensuring the survival of the probiotic, and batch production consistency [275]. A recent meta-analysis of 1551 participants questioned the use of probiotics and prebiotics, as none of them had significant effects on cognitive function. The authors of the study attributed this finding to the low statistical power of the analyzed literature, heterogeneity of the population, cognitive test assessed, and intervention formulation [276]. However, it is also possible that probiotics and prebiotics might not have any effects on cognition at all. It is known that different bacteria present in our gut are capable of synthesizing neurotransmitters and neuropeptides associated with normal human neurotransmission (e.g., catecholamines, histamine, and GABA, among others). The use of certain probiotic bacteria phyla or strains as a therapeutic approach to restore or control the levels of neurotransmitters altered under pathological conditions [236,277] should also consider the impact of ingested bacteria on resident communities (e.g., by directly or indirectly altering either the presence of certain gut bacteria members or production of host-derived molecules) [277].

Prebiotics consist of fibers such as inulin, galacto-oligosaccharides, and fructo-oligosaccharides, among others. Mixed results have been reported with this approach [278,279]. Synbiotics are a combination of probiotics and prebiotics, where prebiotics improve the viability of probiotics [275]. Although limited evidence has been reported, the preliminary results are promising for PD [280]. A recent study shed light on the understanding of the mechanisms used by bacteria to compete with one another for fiber components. This information can be used to more precisely design prebiotic and synbiotic therapeutic approaches that contribute to establishing, restoring, or promoting functions of “healthy” microbiota [281].

Postbiotics are defined as metabolites of bacterial fermentation, including SCFAs. In an experimental mouse model of SCI, oral administration of sodium butyrate, in a dose-dependent manner, has been shown to diminish oxidative stress and inflammation, by reducing NO levels in the spinal cord compared with the control group [282].

Dietary intake, either over the long or short term, can also have an influence on the gut microbiota composition [283]. For instance, a high-fiber diet approach has been associated with enhancing the abundance of *Bacteroides*, *Enterobacteriaceae*, and *Prevotella* spp. compared with the low-fiber diet group in pigs [284]. In humans, an animal-based diet for five days increased the levels of both faecal deoxycholic acid and bacterial genes related to lipid metabolism (i.e., bile salt hydrolases) [283]. A secondary bile acid produced by microbial metabolism was linked to liver cancer [285] and able to impact microbial diversity [286]. It will be of great interest to see how new therapeutic approaches also integrate the effect of diet and gut microbiota on cell metabolism, especially because it has been suggested that cognitive ageing in mice may be reversed by inhibiting PGE2 in myeloid cells [287]. Thus, changes in myeloid cells’ metabolism present a more specific therapeutic target for disorders of aging.

A recent randomized controlled study is open and recruiting SCI patients to study the effect of 8 weeks of a low-carbohydrate/high-protein diet on metabolic function, body composition, quality of life, and gut microbiota for these patients [288]. In a chemically induced PD mouse model, using 1-methyl-4-phenyl-1,2,3,6-tetrahydropyridine (MPTP), a fasting mimicking diet decreased the degeneration of dopaminergic neurons in the substantia nigra and attenuated motor impairment [289].

FMT has been successfully used for the treatment of *Clostridioides difficile* infection, showing not only a high effectiveness, but also safety in humans [290]. The preliminary results of both clinical trials in humans with FMT have been performed in autism spectrum disorders, MS, PD, and AD, showing promising results on neurological symptoms [291,292]. After traumatic SCI in rats, FMT prevented gut dysbiosis and anxiety-like behaviour, without affecting either locomotor recovery or lesion severity [293].

The effect of medication on the gut microbiome, such as the use of antibiotics, statins, metformin, and laxatives, has been associated with microbiome composition [294]. In a mouse model of ALS, antibiotic treatment showed a negative effect on motor readouts [260]. In a mouse model of AD disease, pharmacological treatment with GV-971, a sodium oligomannate restores gut dysbiosis, reduces neuroinflammation in the brain and ameliorates cognitive impairment [240].

The present review has certain limitations that need to be acknowledged. Firstly, it mainly focuses on studies carried out on animal models, but does not comprehensively cover findings from human studies. Secondly, although the field is rapidly changing and we tried to cover the majority of recently published studies, there might have been some of them that we left out from our selection owing to space restrictions.

The communication between the gut microbiota and the brain involves both direct and indirect signalling pathways via chemical neurotransmitters, neuronal pathways, and the immune system. Therefore, therapies aiming to restore gut dysbiosis-inducing pathological conditions should consider that multiple mechanisms and pathways may be acting together to mediate this situation. Importantly, any therapeutic approach to modulate gut microbiota should consider the effect of age, gender, blood cell counts, lipid concentration, exercise, and the host circadian rhythms [287,294,295,296], as all of these factors have been correlated with bacterial composition, diversity, and functional levels. In a recent review, it has been suggested that the first 1000 days in humans (the period from conception to two years of age) would provide a window of opportunity for modulating gut microbiota (e.g., use of probiotics, prebiotics, and FMT, among others) to achieve healthy growth and early childhood development [136]. Of note, new therapeutic strategies should also take into consideration the effects of COVID-19, constant washing of our hands, as well as the use daily cleaning products on our gut microbiota as adults, as well as the effects on the maturation of gut microbiota of children.
